# *In-Vivo* Efficacy of Compliant 3D Nano-Composite in Critical-Size Bone Defect Repair: a Six Month Preclinical Study in Rabbit 

**DOI:** 10.1371/journal.pone.0077578

**Published:** 2013-10-18

**Authors:** Nitin Sagar, Alok K. Pandey, Deepak Gurbani, Kainat Khan, Dhirendra Singh, Bhushan P. Chaudhari, Vivek P. Soni, Naibedya Chattopadhyay, Alok Dhawan, Jayesh R. Bellare

**Affiliations:** 1 Department of Biosciences and Bioengineering, Indian Institute of Technology-Bombay, Mumbai, Maharashtra, India; 2 Nanomaterial Toxicology Group, Council for Scientific and Industrial Research-Indian Institute of Toxicology Research, Lucknow, Uttar Pradesh, India; 3 Regulatory toxicology group, Council for Scientific and Industrial Research-Indian Institute of Toxicology Research, Lucknow, Uttar Pradesh, India; 4 Division of Endocrinology, Central Drug Research Institute (Council of Scientific and 12 Industrial Research), Lucknow, Uttar Pradesh, India; 5 Institute of Life Sciences, Ahmedabad University, Ahmedabad, Gujarat, India; 6 Department of Chemical Engineering, Indian Institute of Technology-Bombay, Mumbai, Maharashtra, India; University of Notre Dame, United States of America

## Abstract

Bone defects above critical size do not heal completely by itself and thus represent major clinical challenge to reconstructive surgery. Numerous bone substitutes have already been used to promote bone regeneration, however their use, particularly for critical-sized bone defects along with their long term *in vivo* safety and efficacy remains a concern. The present study was designed to obtain a complete healing of critical-size defect made in the proximal tibia of New Zealand White rabbit, using nano-hydroxyapatite/gelatin and chemically carboxymethylated chitin (n-HA/gel/CMC) scaffold construct. The bone-implant interfaces and defect site healing was evaluated for a period up to 25 weeks using radiography, micro-computed tomography, fluorescence labeling, and histology and compared with respective SHAM (empty contra lateral control). The viscoelastic porous scaffold construct allows easy surgical insertion and post-operatively facilitate oxygenation and angiogenesis. Radiography of defect treated with scaffold construct suggested expedited healing at defect edges and within the defect site, unlike confined healing at edges of the SHAM sites. The architecture indices analyzed by micro-computed tomography showed a significant increase in percentage of bone volume fraction, resulted in reconciled cortico-trabecular bone formation at n-HA/gel/CMC constructs treated site (15.2% to 52.7%) when compared with respective SHAM (10.2% to 31.8%). Histological examination and fluorescence labeling revealed that the uniformly interconnected porous surface of scaffold construct enhanced osteoblasts’ activity and mineralization. These preclinical data suggest that, n-HA/gel/CMC construct exhibit stimulation of bone's innate regenerative capacity, thus underscoring their use in guided bone regeneration.

## Introduction

New and innovative processes are being evolved to promote and facilitate bone regeneration. Numerous synthetic bone substitutes including metals, polymers and ceramics have already been used to promote bone regeneration. However, their use in bone regeneration, particularly for critical-size bone defects along with their long term *in vivo* safety and efficacy, remains a significant clinical challenge [1–4]. Metals could lead to the induction of inflammatory responses due to their physiochemical interaction with the surrounding tissues, encapsulated polymers exhibit problems in amalgamation with tissues whereas, ceramics are frail and difficult to carve according to defects [4,5]. The most appealing concept in advance and functional biomaterials is to mimic pre-existing composition and structure of the bone. Even more appealing, for early stage repair when bone loss is total or severe, is to mimic the extracellular matrix (ECM) of bone tissue, which establishes a favorable microenvironment for natural repair. Biomaterials having molecular and architectural properties [6,7] that resemble the ECM would result into enhanced integration, biomineralization [8] and tissue in-growth [9], all of which would give rise to strong new bone formation [10–12]. A majority of these processes and techniques involves the application of three-dimensional constructs, particularly blended composites of nano-hydroxyapatite with natural polymer collagen and gelatin (non-immunogenic and degradable derivative of the ECM component collagen) in attempt to mimic the structure of native bone [13,14]. Recently, much consideration has been paid to nano-hydroxyapatite/gelatin (n-HA/gel) composites for their highly osteo-conductive, readily osteo-integrative, easily shaped (injected) and gradual degradation properties [4,15]. Also, several lines of evidence shows that favorable functional groups on polymers incorporated in these composites may carry an important role in concluding bioactivity and calcification properties [16] as they could be expected to be more harmonious with body fluids [17], which further manifest better bone-biomaterial interface and thus ensure *in vivo* endurance [18–20]. As reported previously, the anionic carboxymethyl chitin (CMC) surface may act as a platform for calcium phosphate nucleation (calcification) during bone repair [21–24]. The CMC/HA composite facilitate a high absorbency of blood/exudates which further improves its bonding with host bone [25]. CMC due to its natural abundance, preservative nature and non-cytotoxicity could be used for clinical applications as a stabilizer to its inorganic counterparts used during biomaterial fabrication and functionalization [26–28]. The hybrid materials with controlled and tailorable properties due to favourable ionic interactions between the inorganic and organic constituents could be designed for diverse application in both hard and soft tissue regeneration [29,30]. It would be more effective; if along with the above tailorable hybrid properties the material is carvable and/or amenable to contouring for optimal adaptation to the various shapes of bone defects without failure and or fracture [31–33]. We have developed such biomaterial as a self-organized n-HA/gel/CMC scaffold construct and have been successfully tested concerning its physicochemical, morphological, mechanical, hemocompatible and biocompatible properties to prove the influence of acidic derivative of natural polymer used in functionalization (due to presence of both carboxyl and amino groups) of the composite material [34]. Herein, we test the *in vivo* feasibility of the n-HA/gel/CMC scaffold construct to act as a process-directing agent to promote mineralized tissue formation for the treatment of bone defects.

## Materials and Methods

### 2.1: Preparation of n-HA/gel/CMC composite scaffolds

The n-HA/gel/CMC scaffold constructs were prepared by solvent casting method combined with glutaraldehyde vapor crosslinking and freeze drying method as mentioned in our previous report [34]. In brief, monobasic calcium phosphate is reacted with a calcium hydroxide solution. An acidic premix is formed in first stage by reacting phosphoric acid (Merck, 99%) and analytical grade calcium hydroxide (Merck, 96%) with high shear agitation. Thereafter, the acidic premix was reacted with the saturated solution of calcium hydroxide again under high shear agitation in second stage. The second stage reaction is carried out in an alkaline solution whereby the pH of the solution is maintained at 11 until the complete reaction. It is particularly used as a stoichiometric portion (molar ratio of Ca/P=1.67) of calcium hydroxide in carrying out the reactions. After the hydroxyapatite precipitate is recovered, it is sintered at a temperature of 950°C for about 30 minutes. The powder is ball milled to reduce the average particle size to form a crystalline nano-hydroxyapatite (n-HA). CMC is prepared by chemical modification of chitin powder. Briefly, chitin powder was suspended in 40-50% (w/v) aqueous sodium hydroxide containing a slight amount of sodium dodecyl sulfate under a very low agitation at room temperature and then frozen at -20°C for 12h. The frozen alkaline chitin was then thawed in isopropyl alcohol at room temperature followed by the addition of monochloroacetic acid powder until neutralization at the temperature lower than 15°C. The CMC was then extracted with de-ionized water and salts were removed by dialysis. Gelatin extracted from porcine skin is purchased from sigma Aldrich. Bloom of 300 is selected as to provide more imbibitions of water.

The n-HA (0.5gm) was weighed accurately into a flask, and then deionized distilled water added to make the volume 100ml. The mixture was stirred at room temperature for 2h and ultrasonicated to thoroughly disperse the n-HA powder in the water. CMC (0.5gm) was introduced slowly and the solution was kept under continuous agitation. After stirring over night, 1gm of gelatin was added to the mixture and again stirred for 2h at 37°C. The mixture was then put into the beakers at 4°C to solidify the solvent and induce solvent casting for 2h, and then rapidly transferred to a freezer at -20°C for another 2h. The solidified mixture was maintained at -80°C for 4h and was put inside the liquid nitrogen and then transferred into a freeze-drying vessel for at least 30h (0.036 psi or 0.0025 bars) at -40°C. The dried composite scaffolds were placed on a mesh, in a sealed dessicator containing 25ml solution of aqueous glutaraldehyde (Sigma) for subsequent periods to provide sufficient crosslinking at 40°C. The samples were then retreated with sodium borohydride (NaBH_4_) solution to eliminate the un-reacted glutaraldehyde. Samples were repeatedly washed with and kept for 24h in deionized distilled water and then again the process for lyophilization was repeated. The sample was finally freeze dried for at least 30h at -40°C. To determine a proper crosslinking and controlled degradation, composite scaffolds were exposed in the glutaraldehyde vapor for a timescale of 2h, 4h, 8h, 10h and 12h and then their respective water-resistant/degradation behaviors were evaluated by immersing them in SBF for 14 days. It was found that the n-HA/gel/CMC composite treated in the glutaraldehyde needed 2h to crosslinked sufficiently, which is defined as the retention of structure and integrity when immersed in solution for subsequent period of time. An optimized composition of 0.5gm n-HA, 0.5gm CMC in 1gm gel was found to be the most ideal on the basis of pore size, structural stability and mechanical strength. Composite scaffold of dimensions 15mm high × 3mm wide × 2mm deep, were sterilized with γ -irradiation at 20kGy (or 2.0 MRad) at 30°C in a Gamma Chamber (GC-1200, having ^60^Co as the source) at Tata Memorial Hospital, Parel, Mumbai. The radiation dose given was according to the standards of the International Atomic Energy Agency (IAEA). 

### 2.2: Animals’ procedures

Twenty-eight (28) New Zealand White male rabbits, 06 months old and weighing 1.5-2.0 kg, were used in the study. During the experimental period, the rabbits were held in cages and room temperature and humidity were standardized. The animals were subjected to 12h light/dark cycle and fed *ad libitum* with lucern grass and maintenance diet (Nutrilab rabbit feed, Provimi). The experimental procedure was approved (Animal ethics committee approval no. ITRC/IAEC/34/2010) and conducted in accordance with the Institutional Animal Ethics Committee of Council of Scientific and 12 Industrial Research- Indian Institute of Toxicology Research (CSIR-IITR), as per the guidelines of the Committee for the Purpose of Control and Supervision of Experiments on Animals (CPCSEA), Ministry of Social Justice and Empowerment, Government of India. 

### 2.3: Surgical Methodology

The rabbits were randomly divided into seven groups, based on the duration of the implantation period: group A (01week), group B (02 week), group C (04 week), group D (07 week), group E (10 week), group F (14 week) and group G (25 week) respectively, each consisting of four rabbits. 

#### 2.3.1: Medication

Preoperatively each rabbit was kept off feed for a period of 03h before induction of anesthesia, which was induced by injecting a combination of xylazine (07mg/kg; Intas Pharma Ltd, Ahemdabad, Gujarat, India) and ketamine (60mg/kg; Themis Medicare Ltd, Vapi, Gujarat, India) intramuscularly. The operation sites were depilated and washed/scrubbed with Savlon solution (Johnson and Johnson) prior to surgery.

#### 2.3.2: Defect creation

After the anesthesia, 20mm longitudinal skin incision was made on the dorso-medial surface of the tibia following proper draping of the site. Subcutaneous tissue and periosteum was separated gently from the cortical bone. The periosteum was elevated and retained by a self-retaining retractor. An appropriate defect size of 15mm high × 3mm width × 2mm deep was made using an orthopedic hand drill machine with drill bit size 1.5mm, under constant irrigation with sterile normal saline to avoid thermal necrosis. 

#### 2.3.3: Scaffold insertion

The scaffolds were removed from the sterile patches and wetted with blood that oozed out from the incision during surgery. This helped to make the scaffold compliant. The n-HA-gel-CMC scaffold construct were then placed into the right tibia defect and the left tibial defect remained empty as SHAM to serve as contra lateral controls. 

#### 2.3.4: Wound closure

The periosteum and subcutaneous tissue were sutured with chromic catgut no. 3-0 with simple interrupted sutures. The skin was sutured with nylon, using horizontal mattress sutures. The surgical wound was cleaned with povidone iodine (5%) and dressed with nitro-furazone ointment. 

#### 2.3.5: Post-operative care

An injection of enrofloxacin (05 mg/kg body weight, intramuscularly) was given twice daily for 07 days in order to prevent postoperative infection. An injection of meloxicam (0.1–0.2mg/kg body weight), an anti-inflammatory analgesic, was administered intramuscularly for 03 days postoperatively. The sutures were removed on day 07.

### 2.4: Physical examination

The rabbits were monitored for abnormality of gait. The periods taken for normal weight bearing and ambulation were critically observed in all groups of rabbits. The operated limbs were examined for complications such as swelling, sepsis or pain during the postoperative period.

### 2.5: Gross observations

At the termination of the experiment, animals were euthanized by administering Sodium Pentobarbital ≥100mg/kg IV. The test bones were retrieved and observed for soft tissue reaction around the defect, adhesions, changes in the bone at the site of contact and status of the bone.

### 2.6: Digital radiography

Lateral and anterio-posterior radiographs of the entire lengths of the tibiae were taken preoperatively and immediately after the surgery. Subsequently, radiography of each bone was done on 01 week (group A), 02 week (group B), 04 week (group C), 07 week (group D), 10 week (group E), 14 week (group F) and 25 week (group G) postoperatively. The radiographs were observed for size of periosteal callus, bone healing and complications such as complete fracture of bones and osteomyelitis, if any.

**Figure 1:**
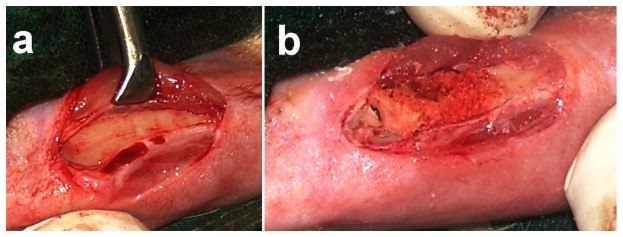
(a) Subcutaneous tissue and periosteum separated gently from cortical bone, and (b) The immediate hemostasis observed at the defect site after application of n-HA/gel/CMC scaffold construct, could helped in the formation of fibrin rich platform which thus, initiated spontaneous healing.

### 2.7: Micro-computed tomography (micro-CT) analysis

Microstructural data quantified from four independent replicates (n=4) for each group. The tibiae were dissected and bone blocks were submerged in a 4% neutral formalin buffered solution, which was changed once after 24h to ensure good fixation. New bone formation in the cortical bone defects and in the trabecular bone area under the defect, where scaffold construct had been placed, was analyzed with a commercially available desktop Micro-CT 1172 scanner (Skyscan, Kontich, Belgium). This system contains an X-ray micro-focus tube of 5µm spot size with high-voltage power supply, a specimen stage with precision manipulator and a two-dimensional X-ray CCD camera. The CCD camera was set with a resolution of 14µm for all the samples. The bone samples were placed on a brass stub with plasticine. Scans were obtained at 100kV and 100µA with the use of an aluminum-copper filter to optimize the contrast, a 360° rotation, an average of 04 frames and a rotation step of 0.4° (2700 images per scan). The reconstruction software (NRecon v.1.4.4) was used to create 2D 2000 x 2000 pixel images. In order to evaluate only mineralized tissue of different densities, the threshold was chosen as the minimum of the histogram of gray levels, programmed into the microCT based on the progression of fracture healing in relation to different ranges of voxel brightness in CTan image analysis software of MicroCT. For consistency, same settings and thresholds were used for treated and contra lateral SHAM groups. The gray-scale images were segmented using a median filter to remove electronic noise. The ring artifact correction was fixed at 12, the smoothing at 01, and the beam-hardening correction at 40%. The region of interest (ROI) was reconstructed from multiple tomography ‘‘slices” for both cortical and trabecular bone. Cortical ROI consisted of a cylinder with a diameter of 03mm and a height of 125 micro-CT slices, starting from the outer cortical bone defect. Trabecular ROI was carefully contoured on the first and the last slice (4mm wide) under the cortical bone defect, while the intermediate slices were interpolated by morphing. Each slice was subsequently visually inspected, and the contour was modified where deemed necessary. ROI of cortical and trabecular bone from each sample was then applied for the analysis of 3D microarchitecture parameters using CTAn software (Skyscan, Kontich, Belgium).

### 2.8: Fluorescence marking

Fluorescent *in vivo* marking with calcein (Cat. No. C0875-5GSigma Aldrich) was performed during the postoperative phase of the study at selected time points to allow analysis of new bone formation as calcein binds to the calcium at newly formed bone surfaces and subsequently on mineralized matrix at fracture site [35,36]. Animals were marked with calcein (50–60 mg/kg body weight) injected subcutaneously, three days prior to autopsy of group A (on day 04), group B (on day 11), group C (on day 25), group D (on day 46), group E (on day 67), group F (on day 95), group G (on day 172). Calcein micrographs were taken from a fixed location and four slides were prepared for each group prior to perform microscopy. Elaborately, each bone was embedded in an acrylic material and 50μm sections were made and the intensity of calcein binding as an indication of the amount of new mineral deposition was calculated taking four such sections (n=4) for each bone, averaged over the entire field of view shown. Calcein is safe for the animals and are incorporated into the bone formed at the time of bioavailability. All rabbits were euthanized and autopsied to collect their tibia for the measurement of bone micro architectural parameters. Bones were embedded in an acrylic material. Sections were made using ‘Isomet bone cutter’ and photographs were taken under confocal microscope (Carl Zeiss LSM 510 Meta) aided with appropriate filters. The intensity of calcein binding calculated using Carl Zeiss AM 4.2 image analysis software.

**Figure 2:**
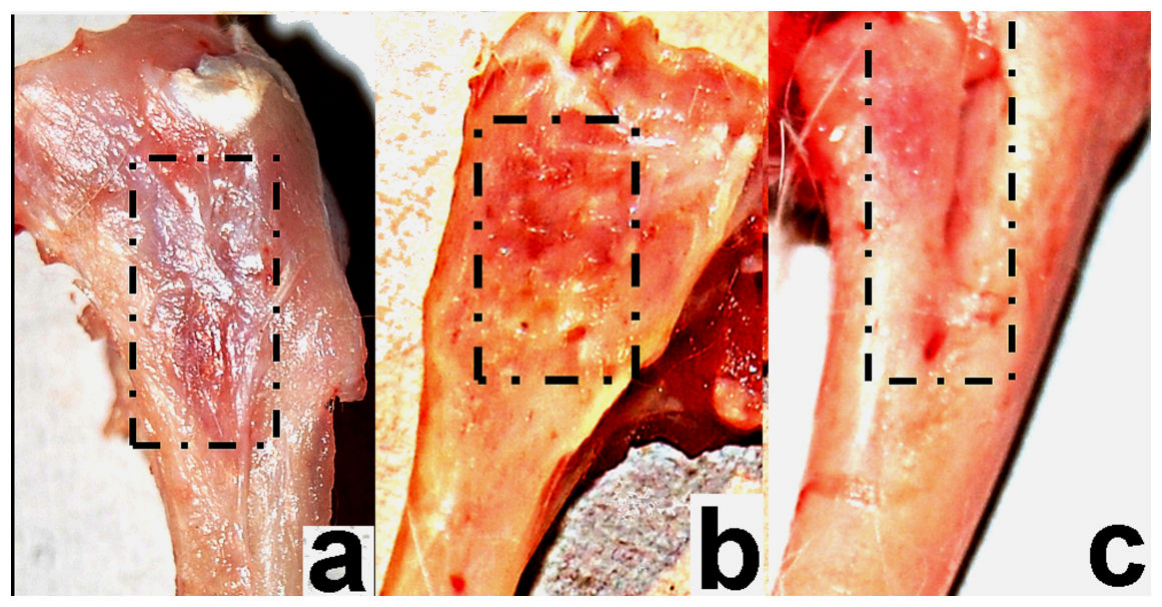
Gross observations of defect site after week 25 (a) treated with n-HA/gel/CMC with adherent soft tissue, (b) after removal of soft tissue, showed ultimate scar-free closure of the cortical window and (c) of SHAM operated site.

### 2.9: Histological studies

Histological examination of the bone was done to evaluate the cellular reactions of the host bone to the implant. The bones from the site of fracture were obtained by cutting them into regular section/piece. The bone section containing defect sites were washed thoroughly with normal saline and fixed in 10% formalin for 07 days. Subsequently, the bone sections were decalcified in 5% nitric acid and checked regularly for the status of decalcification. Once the bone pieces became flexible, transparent and easily penetrable by pins, they were considered to be completely decalcified. The tissues were processed in a routine procedure and 04 μm sections were cut and stained with Hemotoxylin and eosin [37].

### 2.10: Hematological studies

Blood samples of 01ml were collected on respective weeks, before termination of experiments. Hematological parameters were measured using an automated blood analyzer (Sysmex, XT-1800i).

### 2.11: Statistical Analysis

Quantified data are from four independent biological replicates (n=4) is used for each group Data are expressed as mean±SEM unless otherwise indicated. The data obtained in experiments with multiple treatments were subjected to one-way ANOVA followed by Newman-Keuls as post-test analysis. Qualitative observations have been represented following assessments made by three individuals blinded to the experimental designs (*P < 0.05, **P < 0.01, ***P < 0.001). 

## Results and Discussion

### 3.1: Post-operative physical examination

The dose of anesthetic substances, xylazine (07mg/kg) and ketamine (60mg/kg) induced and maintained anesthesia for the creation of bone defects. Animals showed no sign of untoward reaction during the surgical procedure. During the implantation scaffold construct became readily compliant when wetted with blood, which was favorable for surgery. All the rabbits recovered completely within 90-120 min with no post surgery complications and started feeding. No osteolysis, hyperplasia or other adverse tissue responses were observed in the n-HA/gel/CMC scaffold construct or SHAM treated defects throughout the study period of 25 weeks. None of the rabbits showed any abnormality in gait and posture. The surgical wounds healed completely postoperative day 06 and the sutures were removed on day 07 following surgery. There was no evidence of adverse side effects or infection in any of the animals.

### 3.2: Gross observation

Hemostasis at the defect sites was achieved immediately after application of n-HA/gel/CMC scaffold construct (Figure 1b) and maintained during 07 minutes observation period. In the SHAM, hemostasis took longer than 05-07 minutes. The immediate hemostasis at the defect sites after application of n-HA/gel/CMC scaffold construct could help in the formation of fibrin rich platform which by releasing growth factors, initiated spontaneous healing [37–39]. As reported, the platelets are first source of mitogenic factors at a traumatized site to stimulate bone production by releasing platelet-derived growth factor and transforming growth factor beta-1 (TGF-beta-1) [8,40–42]. Also, as detailed in our previous finding n-HA/CMC/gel scaffold construct facilitate the platelet adhesion and hence hemostasis as observed during surgery, could have stimulated initial stage of the repair phase by releasing growth factors, thereby enhancing early stage angiogenesis and bone formation [34]. Post-surgery, all rabbits were healthy with good appetites and showed signs of good healing with no evidence of complications (e.g., hematoma and infection). At the termination of the experiment, the test bones as in Figure 2a, were retrieved and observed for soft tissue reaction around the defect, adhesions, changes in the bone at the site of contact and overall status of the bone. Healing was complete at the defect sites treated with n-HA/gel/CMC scaffold construct at week 25. The n-HA/gel/CMC scaffold construct treated site showed ultimate closure of the cortical window and uniformly filled border of the defect with new bone, resembling host bone as observed in Figure 2b. However in SHAM, as in Figure 2c, the borders of defect site were clearly visible and defined. From the gross observation it is evident that the healing was combined uniformly and the healing rate was faster throughout the n-HA/gel/CMC scaffold construct treated site compared to SHAM.

**Figure 3:**
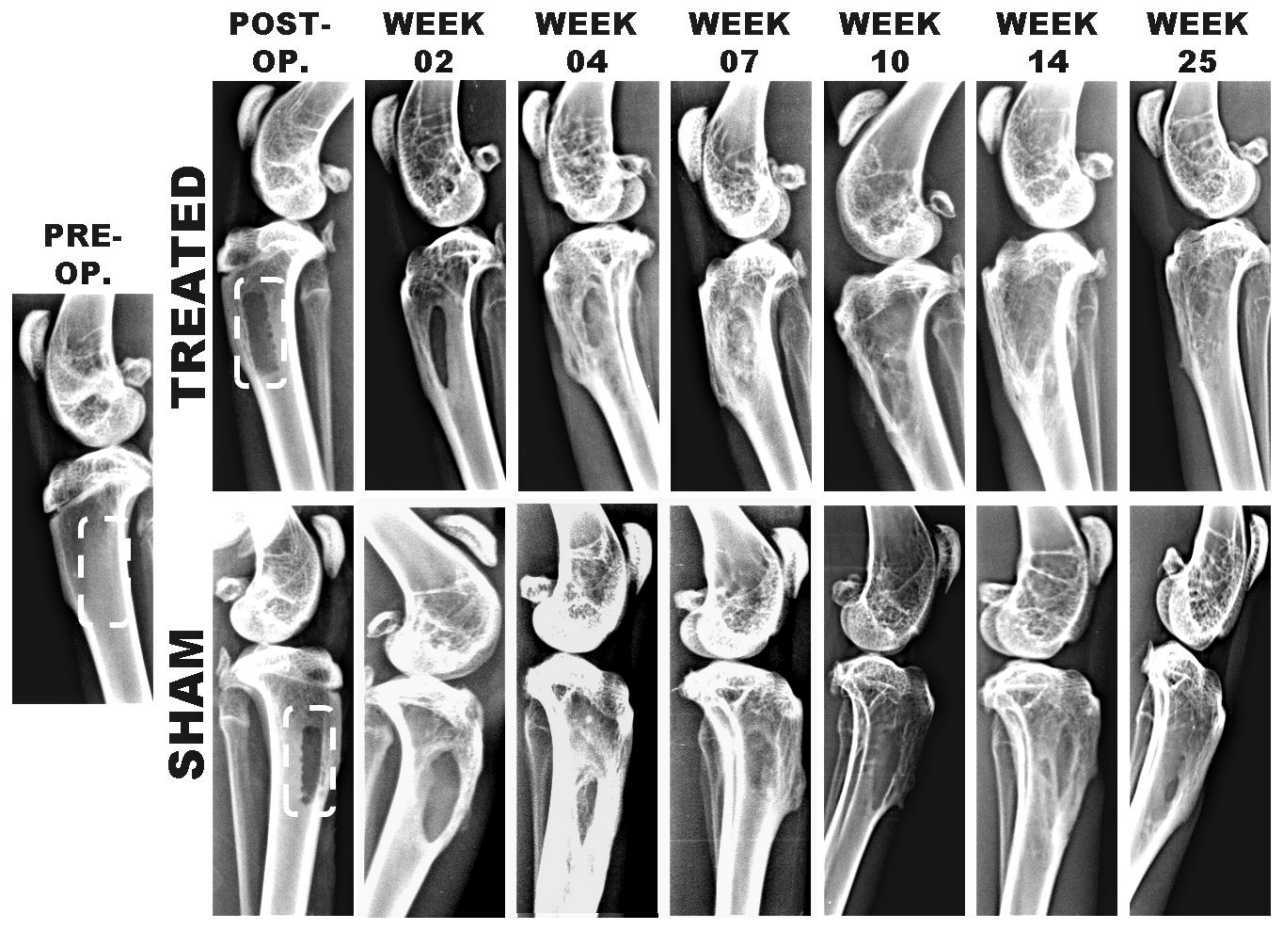
Radiograph of entire length of each tibia, taken pre-operatively (extreme left), immediately after creating defect, and after weeks 02, 04, 07, 10, 14 and 25 of n-HA/gel/CMC treated and SHAM-operated defects. It is worth noting that the radio-density at the n-HA/gel/CMC treated site was comparable to that of the host bone at week 14 and 25.

### 3.3: Radiography

The radiography follow-up showed the first signs of preliminary osteogenesis appeared early in scaffold construct treated site as compared to SHAM. After 02 week, both groups at the defect sites appeared radiolucent to a comparable degree; however after 04 week, the area around the defect site implanted with n-HA/gel/CMC scaffold construct was more radiopaque as compared to SHAM. Radiographs taken on week 07 and 10 showed a well-defined radio-dense area at the defect site treated with n-HA/gel/CMC scaffold construct, due to formation and progression of newly formed bone towards center from peripheral edges. In contrast, bone formation was mainly confined to the defect edges in SHAM treated site. Radiographic evidence in the scaffold construct treated healing areas (Figure **3**) demonstrated that the newly formed bone combined tightly with the adjacent tissue during the course of the study. At the end of week 14 and 25, the increase in the radio-dense area and eventual disappearance of the radiolucent line between the treated implant and the host bone, indicating good bone-implant integration. However, in the SHAM treated sites, the appearance of the border of the defects and presence of voids and gaps at the sites, suggesting incomplete restoration. Unlike soft-tissues, fracture healing is uniquely known to be completed without the formation of scar [40–43]. The constant remodeling of bone tissue provides a mechanism for scar-free healing as mentioned [44]. It is worth noting that during end period of studies as indicated in Figure **3**, the radio-density at the defect site treated with n-HA/gel/CMC scaffold construct was nearly comparable to that of the host bone and is in good agreement with the Figure 2b, which illustrates the ultimate closure of the cortical window after week 25.

**Figure 4:**
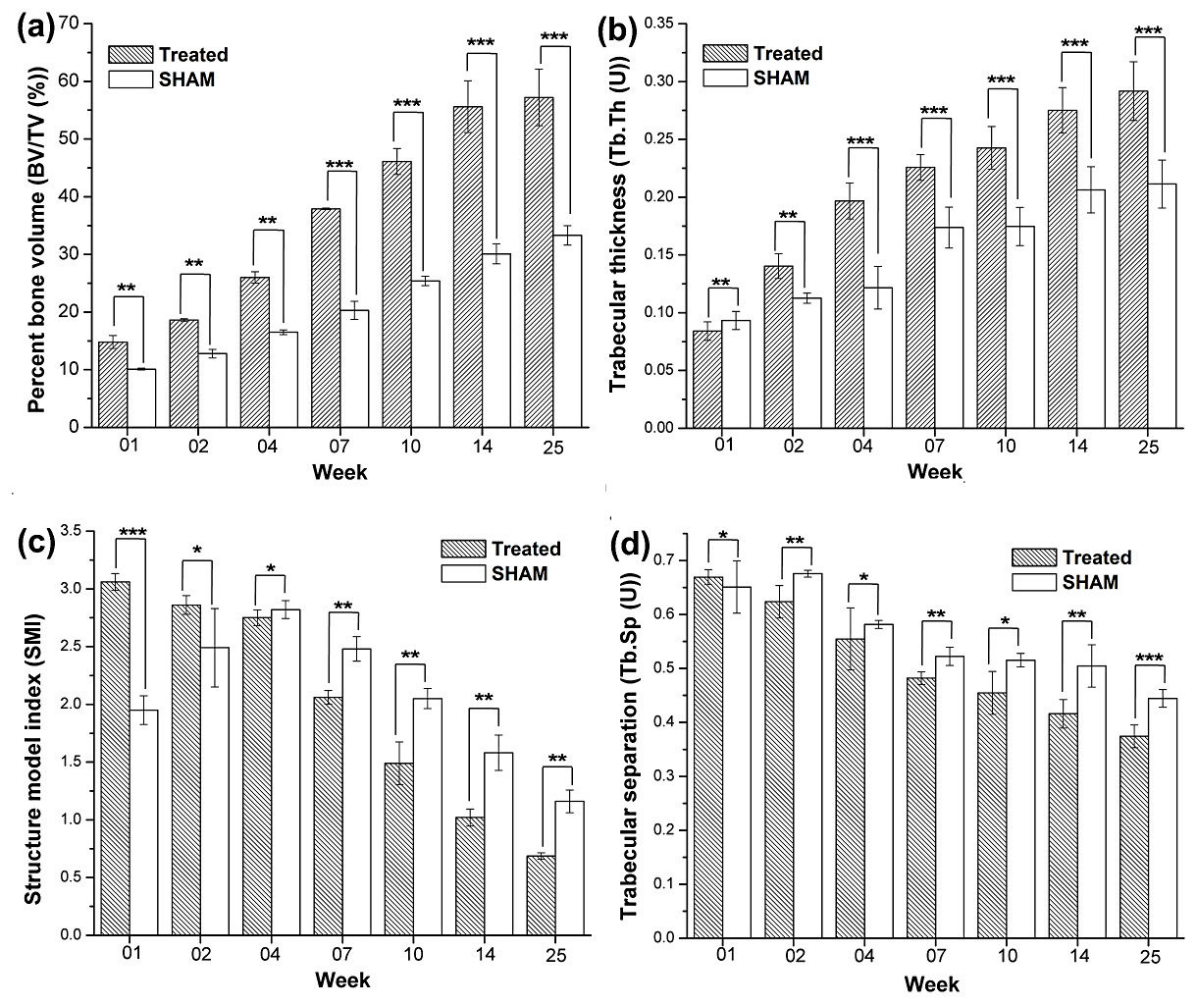
µCT analysis of (a) Percentage of Bone volume/Tissue volume, (b) Trabecular thickness, (c) Structural model index and, (d) Trabecular separation in n-HA/gel/CMC treated and SHAM-operated groups.

**Figure 5:**
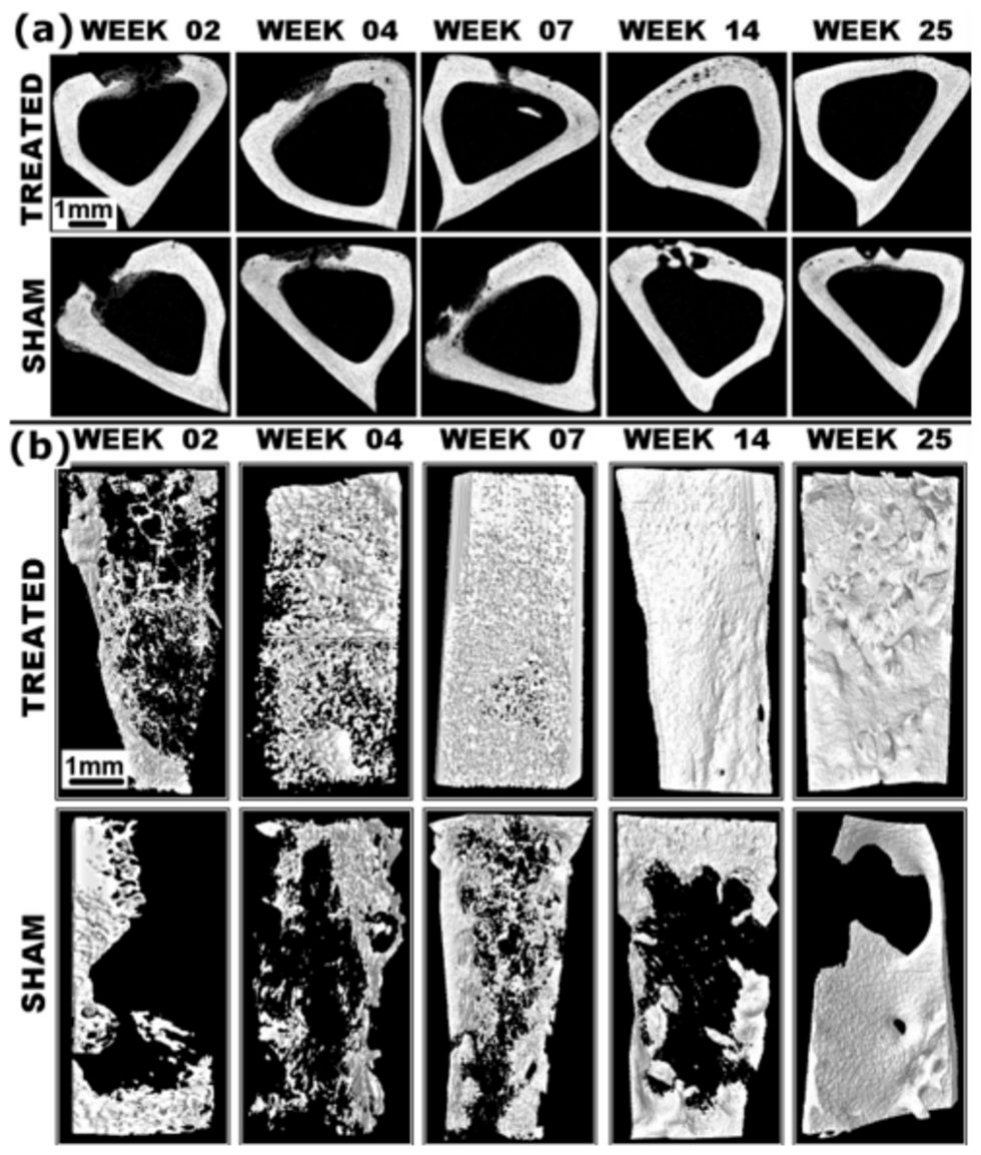
Restored (a) 2-D and (b) 3-D micro-CT images of entire defects (ROI) treated with n-HA/gel/CMC and SHAM-operated defects. Although SHAM defect at week 07 indicated bones’ innate regeneration capacity but due to lack of adequate stability and support to overcome the threshold of physiochemical repair processes, revealed the need of favorable support and environment as provided by n-HA/gel/CMC scaffold constructs for complete reunion at the treated sites. Scale Bar: 1mm.

### 3.4: Micro-CT evaluation of cortical and trabecular bone

Microstructural data quantified from four independent replicates (n=4) for each group, revealed consistent orthotropic bone colonization throughout the study, indicated natural healing process without any inter-fragmentary strain at both the sites. As in Figure 4a and 4b, there was a notable increase in bone volume fraction from 14.8% to 57.2% along with an increased trabecular thickness from 0.0841 to 0.2916 at n-HA/gel/CMC scaffold construct treated sites compared to the increased bone volume fraction from 10.1% to 33.3% and trabecular thickness from 0.0933 to 0.2113 of the SHAM during the study. The predominantly enhanced bone volume and thickness as in Figure **5** demonstrated total interconnectivity and perfect continuity of the newly formed bone network with the trabecular host bone. The structure model index (SMI) as in Figure 4c, quantifies the 3-D bone structure with progression of time, from value 3.06 (perfect rod structure or an infinite circular cylinder) to the value 0.687 (ideal plate structure) while in case of SHAM the values increases to 2.82 up to week 04 showing the random rod like bone formation which then decreased to 1.16 at the end of the study. In case of scaffold construct treated sites the 2D reconstruction (Figure **5**) shows thick structures that seemed to be connected mainly by plates and a small number of rods, which also represented by reduction in trabecular separation (Figure 4d) and SMI. The indices of trabecular separation and structure model index when connected with the BV/TV (%) and Tb.Th. indicating that the architectural transformation from trabecular rods to plates and trabecular thickness was responsible for the bone volume increase in case of scaffold construct treated sites. However, the SHAM restoration indices revealed that the increase in percent bone volume is mainly due to changes in trabecular thickness and less due to architectural transformation from trabecular rods to plates. Structural parameters as well as morphometric and architectural indices, determined from 2D micro-tomographic examination (Figure **5**) at week 14 and 25, indicated complete reunion at scaffold treated site which further dominated by remodeling, whereas SHAM defects still exhibited incomplete restoration at the end of the study.

### 3.5: Fluorescence labeling studies

**Figure 6:**
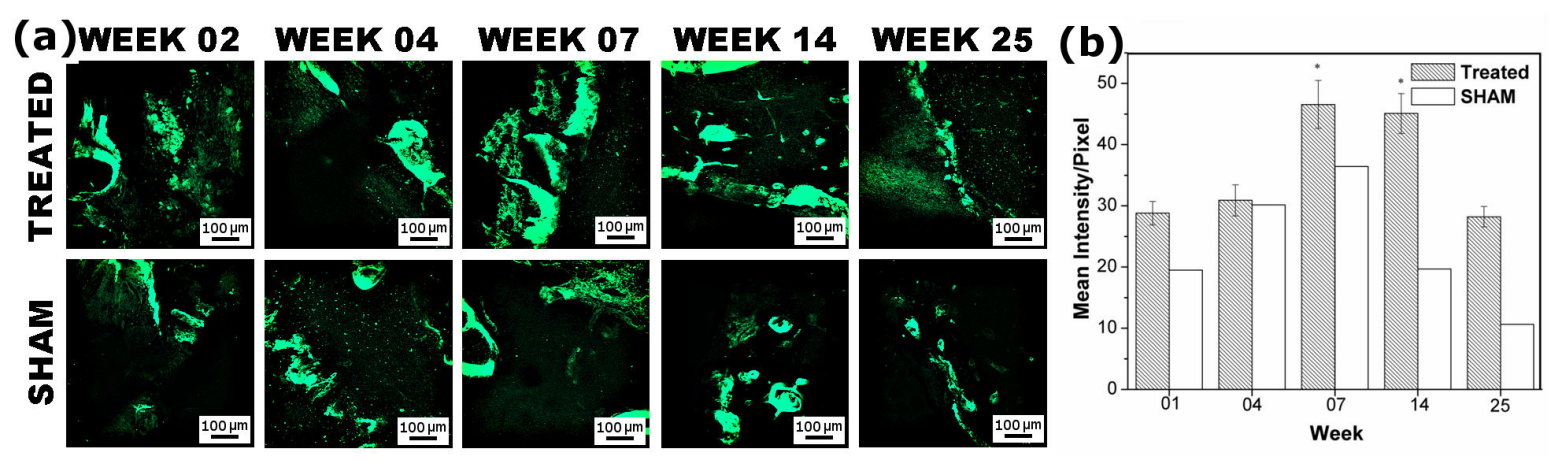
(a) Representative fluorescence images of calcein labeling and (b) graph showing Mean Intensity/Pixel of restored defects treated with n-HA/gel/CMC and SHAM-operated defects. (Scale Bar: 100µm). The absence of significant effect after 14 weeks suggests that most of the healing process was completed and further remodeling overtake new bone formation upto the end of the study.

Fluorescence labeling as in Figure 6a, indicated the initialization of mineralization at week 02. At week 04, more intense and integrated green spots were observed at the n-HA/gel/CMC scaffold construct treated defect sites compared to segregate and diffused green spots in SHAM sites. A well-organized pattern of calcein labeling at week 07 showed extensive formation of lamellar bone, indicating completion of the bone bridging at the n-HA/gel/CMC scaffold construct treated defect sites. This bone bridging further gives the ground support to the bone-forming cells to fill the remaining gaps and voids as revealed by the images at week 14 and 25. Figure 6b shows that the new bone mineral deposition in scaffold construct treated sites increased rapidly from week 02 (28.9±1.9) to week 07 (46.6±3.9) which further remains consistent up to week 14 (45.1±3.3) and then decreased to 28.2±1.6 towards the end of the study. The absence of significant effect after 14 weeks suggests that most of the healing process was complete at that time following which, the remodeling took place until week 25, but no more new bone formation. Similar sequence of events occurred in the SHAM groups except the amount and rate of the new bone formed were respectively lesser and slower than the scaffold construct groups.

### 3.6: Histological investigation

**Figure 7:**
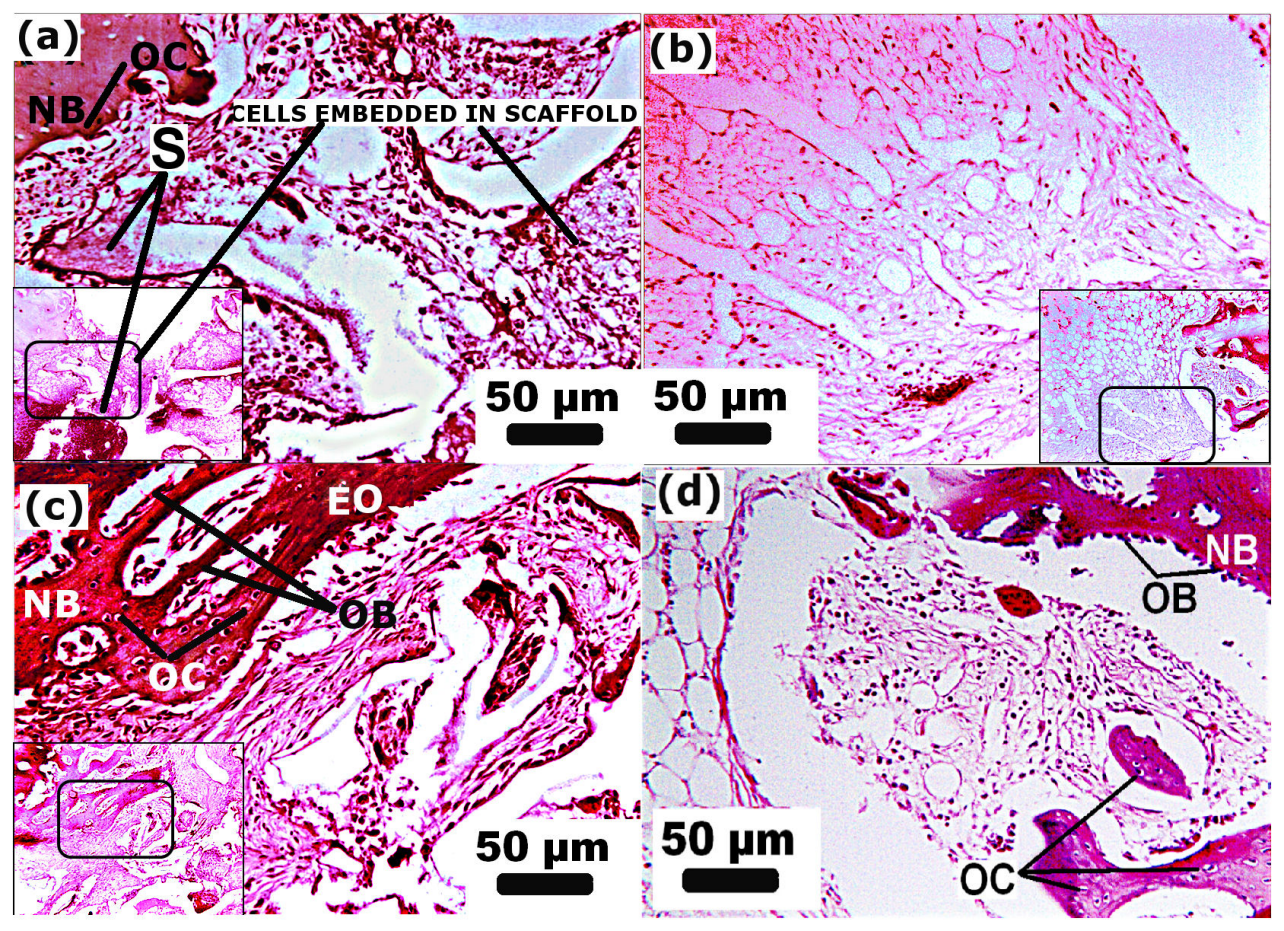
Histology illustrating hematoxylin and eosin (H&E) stained section after week 01 and 02 after implantations of n-HA/gel/CMC (a, c) and of SHAM treated site (b, d) respectively. S: scaffold, NB: new bone, OB: osteoblast, OC: osteocyte. EO: endochondral ossification. Scale Bar: 50µm (magnification=125X, inset magnification=50X).

**Figure 8:**
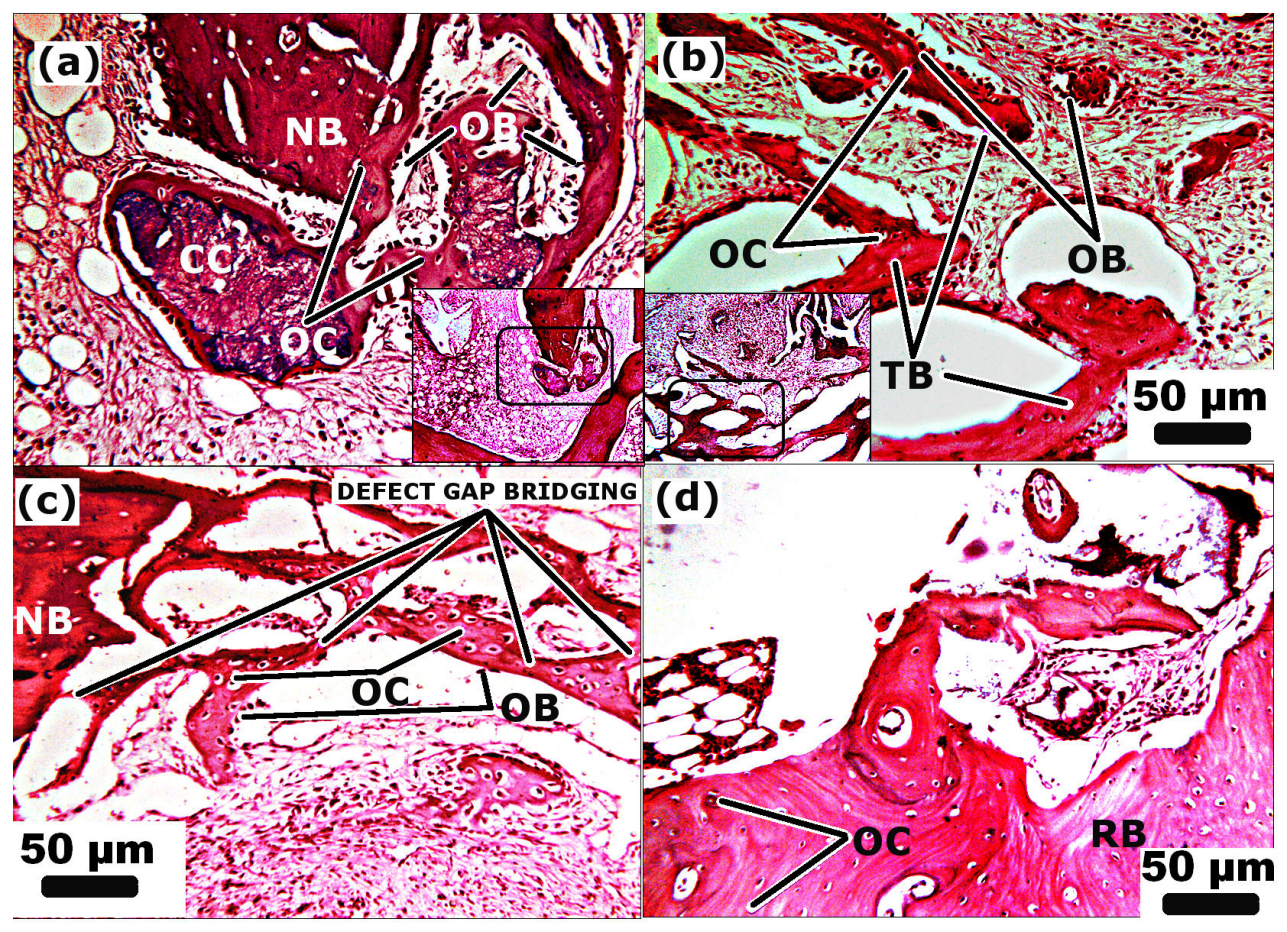
Histology illustrating hematoxylin and eosin (H&E) stained section of n-HA/gel/CMC treated site after week 04, 07, 10 and 14 (a,b,c,d) respectively. Bone forming tissue migrates from periphery to the centre thus initiated bone-bridging. NB: new bone, OB: osteoblast, OC: osteocyte. CC: calcified cartilage, TB: trabecular bone, RB: remodeled bone. Scale Bar: 50µm (magnification=125X, inset magnification=50X).

**Figure 9:**
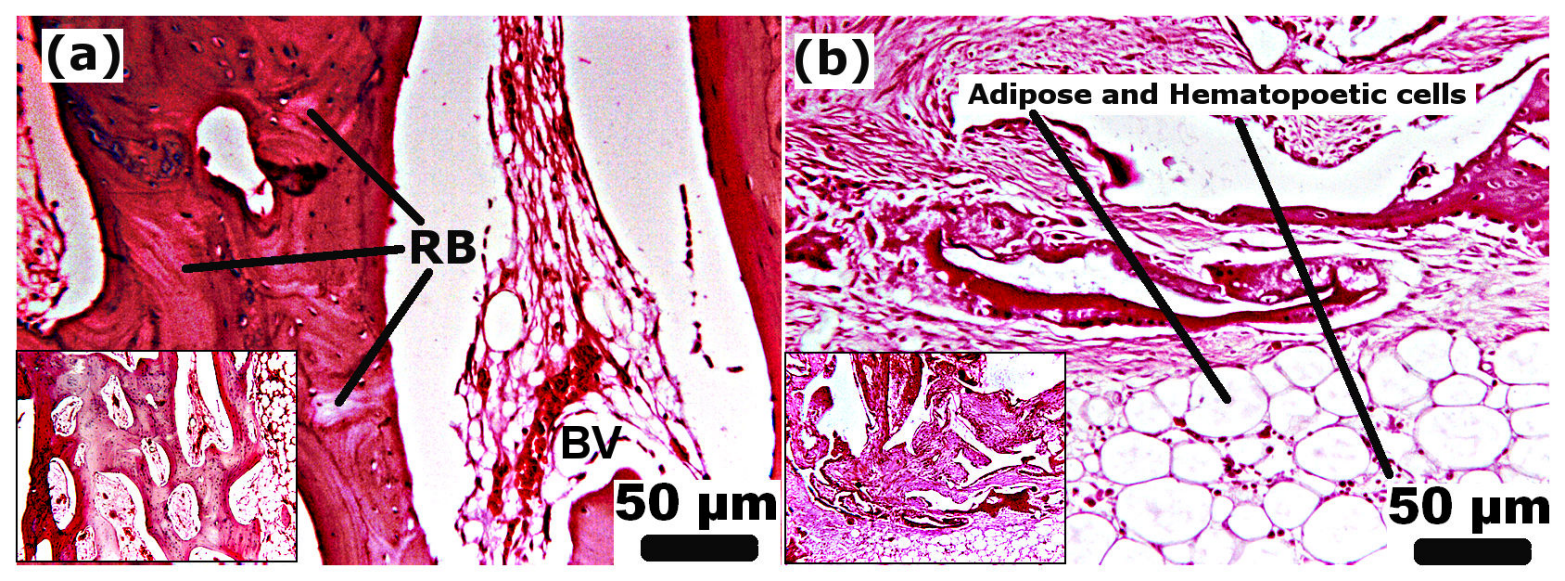
Histology illustrating hematoxylin and eosin (H&E) stained section of (a) n-HA/gel/CMC treated site and (b) SHAM after week 25 respectively. Defect site was covered by mature bone (osteocytes in lacunae) with blood vessel indicated complete remodeling. BV: blood vessels, RB: remodeled bone. Scale Bar: 50µm (magnification=125X, inset magnification=50X).

Histological investigations of the defect site treated with n-HA/gel/CMC scaffold construct and SHAM (Figure **7**, **8** and **9**) were carried out to evaluate its possible suitability as an osteoinductive material. At week 01 postoperatively, granulation tissue and newly formed blood vessels invaded into the micro pores of scaffold as indicated by Figure 7a. Although there was no abundant, a thin layer of new bone (indicated as “NB”) was observed at the peripheral edges. After week 02 as shown in Figure 7c, cuboidal osteoblasts along with granulation tissue began mineralized onto the porous surface of scaffold construct. SHAM showed similar histological changes while with less newly formed bone tissue (Figure 7b and 7d) compared to respective scaffold construct treated sites. With the progression of time, after 04 weeks, as histological characterized by dense matrix deposition, empty spaces were occupied by dense connective tissue with vascular invasion as evident in Figure 8a. Further at week 07 and 10 (Figure 8b and 8c respectively) granulation tissue migrate from periphery to the central region of the scaffold. The formed bone structures grew and began connected, and thus the amount of newly formed bone increased. Many osteocytes in lacunae in mature bone were observed as in Figure 8d, suggested the initiation of remodeling from week 14 onwards. At week 25 as indicated in Figure 9a, the defect treated site was covered by the new bone with lamellar structures invaded with blood vessels while in case of SHAM restoration (Figure 9b) some part of the defect shows remodeled bone with uncompleted healing of the defect site. For an ideal bone formation, an enough stability is prerequisite at the fracture site to keep an interfragmentary strain lower than 2%, to avoid obstruction to medullary vessels and blood flow [40,45,46]. The biomechanical environment and the presence of adequate vascularity at the fracture site determines the repair pattern of the defect [40,47–49]. These requirements may be accomplished by providing the pre-requisite stability and support to the fracture fragments in order to achieve complete bone reunion. Additionally, as reported submicron to macro pore size permit and uphold the cellular activity [50], nutrient flow [51] and bone function [52,53]. According to the previous reports [54,55], scaffolds having small pores (up to 120μm) may have a beneficial effect in initial cell adhesion but ultimately the improved cellular infiltration provided by scaffolds with larger pores (150-450 μm) outweighs this effect. As previously reported by us [34], the n-HA/gel/CMC composite scaffold having regular interconnected pores ranging from diameter of 75μm to 250μm with average pores of 200 ± 40μm) and wall thickness of about 9-12μm. The presence of both small and large pores correlates to their propensity for initial cell adhesion and further stimulation in cellular proliferation respectively In the same context, histology of the bone section in early weeks showed invasion of blood vessels and granulation tissue into the interconnected pores of scaffold construct due to presence of optimal pore size. With further progressive weeks, the histology shows the irregularly oriented osteons which gradually replaced by lamellar bone oriented in the longitudinal axis of the defect cavity.

### 3.7: Hematological studies

Hematology studies are carried out to determine surgery-associated infections during implantation [56]. The results (Table S1) showed no significant difference (p>0.05; ANOVA) in the total Hemoglobin (Hb), erythrocyte and leukocyte count levels between the groups. There was no loss of blood during either surgery or postoperative care. The rabbits in this study were given an anti-inflammatory analgesic following surgery, so the leukocyte count would have remained unaltered during postoperative care. This indicates that surgery and scaffold implantation did not evoke any inflammatory response due to infection. 

The new bone formation, remodeling and cortical reunion at n-HA/CMC/gel scaffold constructs treated sites was superior to its contra lateral empty SHAM treated sites.

## Conclusion

In this study, we assessed the rate and pattern of inner biological development in critical size defects of rabbit, treated with a-cellular n-HA/gel/CMC scaffold construct when compared with self-healing of its empty contra lateral SHAM. In this approach the scaffold construct may offer a favorable platform which induces the ECM through its architectural and chemical cues to use the scaffold construct as a template for faster neo-bone formation by mimicking native bone matrix as shown in Figure S1. The uniformly interconnected pores and anionic nature of these scaffold constructs are substantial in determining the flow-pattern of body-milieu and thus can activate “contact guidance,” which further facilitates the influx and synchronization of bone forming host cells. This may be due to strong interaction of Ca^2+^, PO_4_^3-^ of n-HA and free NH_4_^+^ and COO^-^ groups of gel/CMC polymers. The viscoelasticity (Video S1) of these scaffold constructs in wet condition i.e., mixing these materials with autogenous blood/body milieu makes it capable of being withstand cyclic loading without failure/fracture. Additionally, the viscoelastic nature of the scaffold constructs by its convenience; this resilient scaffold construct is spongy and therefore it does not require the tedious and time consuming need for accurate carving that most other bone substitutes suffer from. Hence it can be use off-the-shelf by the surgeon into the patient. The n-HA/CMC/gel scaffold constructs can be squeezed to fit according to bone defect/cyst and large defects with smaller opening with negligible force being exerted on the defect walls, and thus, improve the initial ability to integrate with living tissues, without unexpected breakage/formation of debris. Furthermore, due to abundance of natural polymers i.e., gelatin and carboxymethyl chitin, the n-HA/CMC/gel scaffold construct sounds to be very socioeconomic as a bone regenerative material for critical fractures. The speed and quality of bone ingrowths’ and bone remodeling observed at the defect site treated with the n-HA/gel/CMC construct, underscoring their use as a 3D scaffold for clinical applications, particularly in orthopaedics, maxio-facial surgery and dentistry.

## Supporting Information

Table S1 **Follow-up results of hematology data of various groups during post-operative care.**
(DOCX)

Figure S1 **SEM micrograph of scaffold construct treated entire defect site after week 10.** The micrograph demonstrates the interfacial interaction of newly formed bone and its structural integration, mimicking old bone. OB: old bone, NB: new bone, IF: interface. Scale Bar: 10 µm.
(TIF)

Video S1 **Scaffold’s viscoelastic behavior (AVI).**
(AVI)
