# Supplementary material for: In-Vivo Efficacy of Compliant 3D Nano-Composite in Critical-Size Bone Defect Repair: a Six Month Preclinical Study in Rabbit
Source: PLoS One. 2013 Oct 18;8(10):e77578. doi: 10.1371/journal.pone.0077578 (PMC3799616; doi:10.1371/journal.pone.0077578)
Supplement: Table S1 — Follow-up results of hematology data of various groups during post-operative care. (DOCX) [file pone.0077578.s001.docx]

**Supporting Information**.

**In-vivo efficacy of compliant 3D nano-composite in critical-size bone defect repair: a six month preclinical study in rabbit**

Nitin Sagar ^1^, Alok K. Pandey ^2^, Deepak Gurbani ^2^, Kainat Khan ^4^, Dhirendra Singh ^3^, Bhushan P. Chaudhari ^3^, VivekP. Soni ^1^, Naibedya Chattopadhyay ^4^, Alok Dhawan ^2, 5^, Jayesh R. Bellare ^1, 6,*^

**Table S1.** Follow-up results of hematology data of various groups during post-operative care.

| **Parameters►**  **Groups▼** | **WBC**  **10^3^/µL** | **RBC**  **10^6^/µL** | **HGB**  **g/dL** | **HCT**  **%** | **MCV**  **fL** | **MCH**  **pg** | **MCHC**  **g/dL** | **PLT**  **10^3^/µl** | **NEUT**  **%** | **LYMPH**  **%** | **MONO**  **%** | **EO**  **%** | **BASO**  **%** |
| --- | --- | --- | --- | --- | --- | --- | --- | --- | --- | --- | --- | --- | --- |
| Control | 5.8±0.9 | 6.12±0.7 | 13.0±1.0 | 40.2±3.2 | 65.9±2.4 | 21.3±0.8 | 32.3±0.3 | 172.0±27.1 | 30.4±6.9 | 58.3±10.3 | 7.3±2.1 | 2.0±0.3 | 2.1±1.5 |
| Week 01 | 4.9±0.1 | 7.09±0.1 | 13.1±1.3 | 42.0±4.2 | 59.4±4.3 | 18.5±2.2 | 31.2±0.1 | 144.5±15.1 | 26.8±6.8 | 58.5±4.6 | 8.2±0.2 | 2.0±0.1 | 4.6±0.1 |
| Week 02 | 4.8±0.3 | 6.41±0.2 | 13.6±0.2 | 44.9±0.1 | 70.2±0.4 | 21.4±0.4 | 30.4±0.4 | 188.5±4.2 | 34.9±2.3 | 55.2±0.4 | 6.7±0.5 | 1.5±0.1 | 1.9±0.1 |
| Week 04 | 7.0±0.5 | 6.69±0.1 | 13.9±1.6 | 43.6±4.3 | 65.5±3.2 | 20.9±0.6 | 31.9±0.6 | 252.0±4.6 | 20.9±3.1 | 68.4±5.2 | 7.2±0.4 | 1.0±0.1 | 2.55±0.1 |
| Week 07 | 7.0±0.4 | 6.86±0.3 | 14.2±0.8 | 44.8±2.8 | 65.4±0.5 | 20.7±0.3 | 31.7±0.1 | 212.0±13.9 | 23.4±2.8 | 64.6±2.1 | 7.7±0.6 | 1.6±0.1 | 2.8±0.1 |
| Week 10 | 6.5±0.4 | 7.80±0.1 | 14.3±0.2 | 45.2±2.1 | 58.9±1.2 | 18.6±3.1 | 31.7±0.2 | 266.0±7.9 | 21.9±5.1 | 67.5 ±4.9 | 7.0±0.2 | 1.9±0.1 | 1.85±0.1 |
| Week 14 | 8.1±0.2 | 6.97±0.1 | 14.8±0.7 | 47.0±2.4 | 67.5±4.5 | 21.3±1.3 | 31.5±0.1 | 227.0±5.7 | 23.1±4.2 | 66.1±4.3 | 8.3±0.2 | 0.8±0.1 | 1.75±0.1 |
| Week 25 | 7.1±0.1 | 7.28±0.2 | 14.6±1.1 | 45.8±0.2 | 62.9±0.1 | 20.1±0.1 | 31.9±0.1 | 145.0±2.8 | 22.4±2.2 | 66.4±1.3 | 5.6±0.7 | 3.6±0.2 | 2.05±0.1 |
