# Supplementary figures and images for: In-Vivo Efficacy of Compliant 3D Nano-Composite in Critical-Size Bone Defect Repair: a Six Month Preclinical Study in Rabbit
Source: PLoS One. 2013 Oct 18;8(10):e77578. doi: 10.1371/journal.pone.0077578 (PMC3799616; doi:10.1371/journal.pone.0077578)

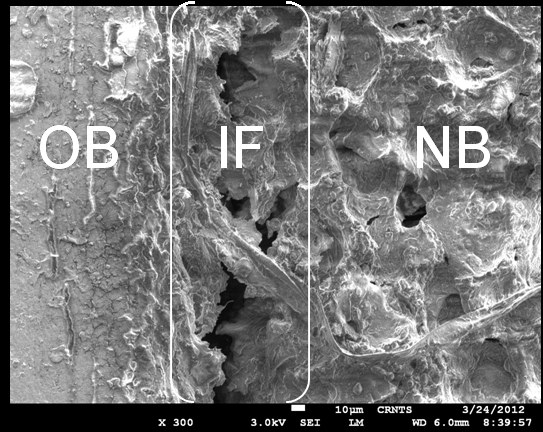

Supplement: Figure S1 — SEM micrograph of scaffold construct treated entire defect site after week 10. The micrograph demonstrates the interfacial interaction of newly formed bone and its structural integration, mimicking old bone. OB: old bone, NB: new bone, IF: interface. Scale Bar: 10 µm. (TIF) [file pone.0077578.s002.tif]
